# Harnessing Nature’s Toolbox: Naturally Derived Bioactive Compounds in Nanotechnology Enhanced Formulations

**DOI:** 10.1021/acsomega.4c07756

**Published:** 2024-10-18

**Authors:** Chetana Sanjai, Santosh L. Gaonkar, Sushruta S. Hakkimane

**Affiliations:** † Department of Biotechnology, Manipal Institute of Technology Bengaluru, Manipal Academy of Higher Education, Manipal, Karnataka 576104, India; ‡ Department of Chemistry, Manipal Institute of Technology, Manipal Academy of Higher Education, Manipal, Karnataka 576104, India

## Abstract

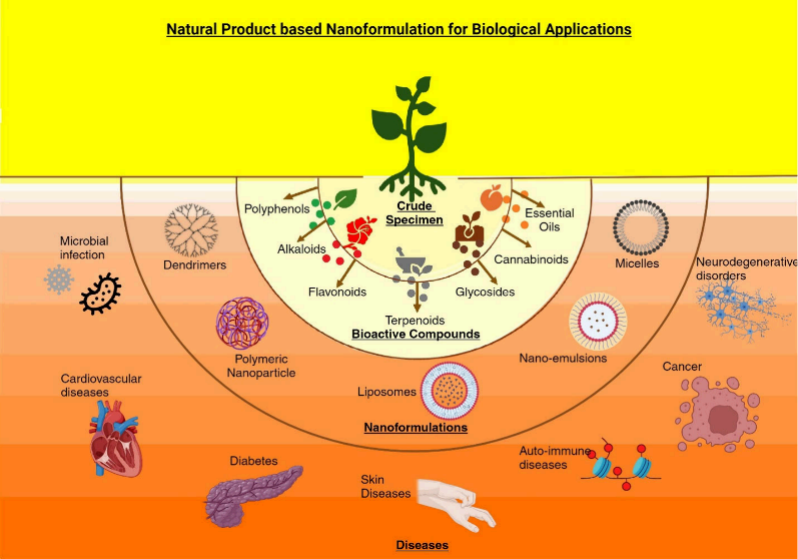

The vast diversity of plants in nature offers a rich reservoir of bioactive compounds that have historically played an integral role in pharmacotherapy and continue to serve as a primary source of novel therapeutic agents. Medicinal plants contain a multitude of secondary metabolites with pharmacological potential, making them indispensable in drug discovery and development. These bioactive constituents, inherent in herbal remedies, exhibit a wide range of medicinal properties due to their complex chemical compositions and structural diversity. Despite their therapeutic potential, the clinical application of crude plant extracts is often hindered by limitations, such as poor bioavailability, low biostability, and variable efficacy. These issues can diminish the therapeutic impact of plant-derived compounds. Nanotechnology presents an innovative approach to addressing these challenges through the development of nanoformulations that enhance the efficacy of bioactive compounds. This review examines both historical and recent studies on the synthesis and characterization of bioactive compounds, focusing on their effectiveness in treating various diseases. Additionally, it addresses the risks associated with the direct use of crude plant extracts in medicine, explores extraction and isolation techniques, and reviews research from the past five years on the development of bioactive compounds, their nanoformulations, and their applications in disease treatment. The review also presents recent clinical trials conducted over the last five years on crude extracts and their nanoformulated counterparts, providing insights into the clinical translation of these natural therapeutics.

## 1 Introduction

Natural products (NPs) have historically been important to humankind as a source of medicinal drugs but also pose challenges for drug discovery and development. In India, China, Latin America, Africa and Caribbean, plants have traditionally been utilized as medicines.^1,2^ NPs (also called secondary metabolites) have transformed contemporary pharmaceuticals and hold great potential for both illness prevention and treatment.^3^ They contain powerful antioxidants with anti-inflammatory properties.^4^ NPs, however, offer a distinct structural variety compared to conventional combinatorial chemistry, which provides chances to find mostly new low-molecular-weight lead molecules.^5^ A vast reservoir of untapped natural lead compounds is yet to be discovered, as less than 1% of the world’s biodiversity has been thoroughly investigated for potential biological activity.^6^ This suggests that many more valuable natural compounds with beneficial properties remain undiscovered. Recently, clinical investigations have focused on NPs, especially antimicrobial and anticancer drugs.^5,7^ Although natural items are typically overlooked, compound libraries are being created in part to resemble the chemical features of NPs more accurately. NPs have long been recognized as key participants in the drug development process, particularly in the areas of infectious and cancer therapies but also in the fields of cardiovascular, rheumatoid, diabetic, multiple sclerosis, and neurodegenerative illnesses.^8−10^ The crude extract derived from NPs is composed of many bioactive components. These bioactive compounds can inhibit certain infections biologically. So far, several plant-derived secondary metabolites have been identified, each with a distinct pharmacological profile and structure.^11^ When Fleming unintentionally discovered penicillin in 1929 from the filamentous fungus *Penicillium notatum* and observed its widespread therapeutic application in the 1940s, the “Golden Age” of antibiotics in medicine officially began. This discovery also prompted extensive studies on nature as a potential source of novel bioactive compounds.^12^ The use of a thousand distinct herbs as medicine was documented in an ancient collection of Ayurvedic hymns dating back to 1000 BC, providing evidence of plant-based treatment in India.^13^ The development of contemporary medicine relies on scientific knowledge and scientific observational efforts. However, a large portion of this knowledge was developed from our ancestors’ ancient knowledge. To date, many novel natural compounds that have been used for curing several diseases have been discovered. The advancement of omics has facilitated the use of NPs in the development of new drugs. However, the development of herbal extracts is limited by their low availability, complex structure, poor solubility, and lack of a mechanistic understanding. Therefore, new strategies and technologies are needed to overcome these hurdles and explore the full potential of NPs as therapeutic agents. One of the strategies to overcome poor solubility, bioavailability, stability, and targeted delivery are the combination of pharmacognosy and nanotechnology. NPs are more willingly absorbed than synthesized medications, according to research.^14^ By enhancing the targeted administration of drugs, reducing side effects on nontargeted organs, and increasing the solubility and bioactivity of pharmaceutical compounds, nanotechnology can assist them in improving their pharmaceutical features. Nanostructured materials can be developed as inorganic nanoparticles (NPs), nanotubes, nanofibers (NFs), and liposomes to overcome the shortcomings of pharmaceuticals and transport therapeutic substances to the target sites in an effective manner. Over the past few decades, there has been a rapid development of nanomedicines; some have been licensed by the FDA and utilized as first-line anticancer medications. For instance, the paclitaxel (PTX)-loaded human albumin particle formulation Abraxane has been approved to treat metastatic breast cancer because of its tumor-targeting properties and antitumor efficacy. Its capacity to target tumors and fight cancer is stronger than those of formulations without PTX.^15^ Various agents are employed to stabilize the active agent and improve its bioavailability in the medical domains of environmental sustainability, biosensing, imaging, theranostics, and cancer, where nanoparticles have interesting applications as well. As different agents are employed to stabilize the active agent and increase its bioavailability, nanoparticles also show promise in the medical disciplines of environmental sustainability, biosensing, imaging, theranostics, and cancer.^16^ This review presents the bioactive potential, risks related to crude extract, a glance into the mechanism in the human body, and role of nanotechnology and nanoformulation of crude extract in the last five years.

## 2 The Bioactivity Potential of Natural Products

The majority of the current research on the biological activity of NPs concerns different plant species. Natural chemicals take up a large portion of plants and have antimicrobial, antiparasitic, immunostimulant, anticancer, anti-inflammatory, antioxidant, hepatoprotective, and neuroprotective effects. Additionally, they also detail novel applications for the actions of medicinal herbs that are either fully known or partially known. The plant world is still a vastly untapped source of abundant pharmaceutically and industrially important NPs. We can also derive highly valuable biologically active substances from other organisms, such as microbes, fungi, and animals (such as insects).^17^ Various studies have been carried out on multiple plants, revealing their ability to inhibit cancer and microbial activity, induce tumor-suppressing autophagy, and promote cytoprotective autophagy^18^ in diabetes,^19,20^ and neurodegenerative diseases.^10^ In the Chinese tradition, the widely used plants are *Taraxacum officinale, Coptis rhizome*, and *Scutellaria baicalensis*(21) and in Ayurveda, several plants are renowned for their antibacterial properties, such as Tulsi, tamarind, garlic, neem, turmeric, cinnamon, aloe vera, Indian gooseberry, and Triphala (a combination of three fruits).^22,23^ The beverages used daily, such as tea *C. sinensis*, contain nonpolymeric constituents and polymeric tannins, which are major constituents that contribute to the antioxidant and antibacterial properties of green, black, and herbal tea.^24^ Chamomile,^25^ Jasmine,^26^ ginger, and white tea^27,28^ have antimicrobial, antiaging, anticancer, neuroprotective, and antioxidant effects.^29,30^ Red wine is mostly composed of polyphenols and is recognized for its anticarcinogenic and cardiovascular-protective properties. Numerous studies have examined the health benefits of red wine polyphenols on human gut microbiota, cardiovascular disease, cancer chemopreventive activities, neuroprotective effects, and other areas. These findings are elucidated in refs (31,32). Similarly, many different natural sources, such as pepper, beetle leaves, ginger, aloe vera, cranberry, and echinacea, contain various phytochemicals that enable them to combat bacteria, fungi, and malignant cells. Latin America, being rich in biodiversity, has a rich history of bioactive compounds like curare, cocaine, quinine, and capsaicin contributing to drug discovery and development.^33^

## 3 Advances in the Use of Natural Products as Therapeutic Agents

NPs are a broad class of chemical entities that are the source of active compounds. Many studies are being conducted to determine and isolate the active ingredients that facilitate many uses in healthcare (human and veterinary) and agriculture. NPs have been the oldest area of investigation since the 1800s, and their investigation continues to be the most researched area. Morphine, the initial active compound found in *Papaver somniferum* pods, was identified by Li and Vederas in 2009.^34^ Acetylsalicylic acid, popularly known as aspirin, was discovered in 1852 by Charles Frederic Gerhardt and later synthesized by Felix Hoffmann in 1859. Gerhardt initially described the reaction between sodium salicylate and acetyl chloride, resulting in crude acetylsalicylic acid. Although Gerhardt mentioned this discovery in his 1853 publication “Research on anhydrous organic acids,” it gained widespread recognition when Felix Hoffmann successfully identified and synthesized acetylsalicylic acid in 1897.^35^ Since then, various approaches for synthesizing acetylsalicylic acid have been explored, including the use of solid acid catalysts such as nanocrystalline sulfated zirconia^36^ and the reaction between o-hydroxy salicylic acid and acetyl chloride. These methods have facilitated the development of efficient and environmental friendly routes for acetylsalicylic acid synthesis. Curcumin, which is extracted from the rhizome of *Curcumin longa*, is the most widely utilized yellow coloring agent worldwide. Turmeric, subsequently known as Curcumin, gained significant recognition in the early 19th century and was initially identified by Vogel and Pelletier in 1815; its chemical structure was subsequently elucidated by Milobedzka and Lampa. Renowned for its anti-inflammatory, antioxidant, and anticancer properties, curcumin exhibits minimal toxicity and promising potential.^37,38^ For generations, the Chinese population has utilized artemisinin as a medication to treat fever and malaria. Tu Youyou, a Chinese scientist who oversaw a covert study in the 1960s and 1970s to find a treatment for malaria, is credited with the discovery of artemisinin. After screening millions of herbal extracts, Tu and her team discovered that an extract *from Artemisia annua* had strong antimalarial properties. The active component was subsequently separated and refined, and it was given the name qinghaosu, which translates to a “sweet wormwood element”.^39^ In 2015, the discovery and advancement of this substance earned it the Nobel Prize in Physiology or Medicine. Since 1871, Echinacea has been employed as a remedy for various ailments, as initially noted by Kh.K.F. for its purported blood-cleansing properties. Mayer, an untrained individual, publicly allowed a snake to bite him while simultaneously using Echinacea to purify his blood. This incident sparked the researcher’s profound interest in the plant, prompting extensive investigations thereafter.^40^ By the late 19th and early 20th centuries, Echinacea had become the most favored herbal remedy in America. Dr. Gerhard Madaus, a German physician, introduced Echinacea to Europe during the 1930s and conducted extensive research on it is pharmacological properties and therapeutic applications.^41^ In 1928, Alexander Fleming discovered a fungus belonging to the genus *Penicillium* that inhibited the growth of bacteria.^42^ In the most recent discoveries, a novel antibiotic named Teixobactin, which is produced by an undescribed soil microorganism that kills Gram-positive bacteria, was discovered in 2015.^43^ Vinca alkaloids, which include vinblastine and vincristine, were first obtained from *Catharanthus roseus G. Don. (*Madagascar periwinkle plant). Plants like *Uncaria tomentosa* (cat’s claw) and *Paullinia cupana* (guarana), which have long been used in traditional medicine, are presently being researched for their ability to treat a range of ailments.^44,45^ Renowned for their potent antitumor properties, these alkaloids are frequently integrated into combination chemotherapy protocols for breast cancer treatment. Over the years, derivatives such as vinorelbine and vinflunine, which are generated through semisynthesis, have exhibited anticancer efficacy. Extensive clinical trials have scrutinized the effectiveness of vinca alkaloids against breast cancer, establishing their role as a standard treatment for more than three decades. Currently, derivatives such as vindesine and vinorelbine are widely used in clinical settings, while vinflunine is undergoing phase III clinical trials.^46,47^ At present, there has been an increase in the percentage of clinical trials assessing the efficiency and safety of NPs. These trials provide valuable data on the potential of NPs as therapeutic agents (Table 1). Advances in technology, such as high-throughput screening, metabolomics, and bioinformatics, have enabled the efficient identification, isolation, and characterization of bioactive compounds from natural sources.^48^ Moreover, there is a growing appreciation for the synergistic effects and complex interactions found in NP mixtures, leading to the development of novel combination therapies.

**Table 1:** Clinical Trial of Natural Products from Plants

**Condition Targeted**	**NCT Number**	**Intervention**	**Phase**	**Status**
Breast Cancer	NCT05296577	• Anlotinib and vinorelbine	Phase 2	Recruiting
		• Vinorelbine injection		
Rhabdomyosarcoma	NCT04299113	• Vinorelbine	Phase 1	Recruiting
		• Mocetinostat		
Breast Cancer	NCT05823623	• Inetetamab	Phase 2	Recruiting
		• Pyrotinib		
		• Oral Vinorelbine Tartrate		
Breast Cancer	NCT05747326	Oral vinorelbine and capecitabine	Phase 2	Recruiting
In Utero Drug Exposure	NCT04050189	• NPs: Probiotics	Phase 2	Completed
		• Placebo		
Sjogren’s Syndrome Xerostomia	NCT04252209	• Natural herbs of coconut, aloe vera, and peppermint	Phase 3	Completed
		• Carboxy methyl cellulose		
Skin Condition	NCT05310994	• Placebo drink	Not applicable	Completed
		• Wasabi Leaf extract Drink		
Photoaging Hyperpigmentation Rhytide	NCT04586816	• 1% red maple leaf extract in a cream base.	Not applicable	Completed
		• 5% red maple Leaf extract		
		• Vehicle		
Diabetes Mellitus, Type 2	NCT05605704	Atherolive	Phase 2	Recruiting
			Phase 3	
Hypertension	NCT05636826	Atherolive-drug	Phase 2	Not yet Recruiting
			Phase 3	
• Motoric Cognitive Risk Syndrome	NCT04492241	• Ginkgo Leaf Extract and Armillariella Mellea Powder Oral Solution.	Not applicable	Recruiting
• Mild Cognitive Impairment		• Simulation of Ginkgo Leaf Extract and Armillariella Mellea Powder Oral Solution		
• Aging				
• Locomotive Syndrome				
Periapical Abscess	NCT02943759	• Neem leaf extract.	Phase 2	Completed
		• Chlorhexidine gluconate	Phase 3	
Dengue	NCT06121934	• Carica Papaya leaf extract.	Phase 3	Completed
Rheumatoid Arthritis	NCT05665985	• *Moringa oleifera*	Phase 1	Completed
			Phase 2	
Necrotic Pulp	NCT05348824	• Dietary Supplement: *Moringa oleifera* leaf	Phase 2	Not yet Recruiting
		• Sodium hypochlorite	Phase 3	
Smoking Cessation	NCT06091826	• NFL-101	Phase 2	Completed
• Nicotine Dependence				
• Cardiovascular Diseases	NCT05504044	• Glucose control	Not Applicable	Completed
• Metabolic Disease		• Bread control		
		• Almond paste		
		• Almond paste and inulin		
		• Low dose almond paste and inulin		
• Crohn Disease	NCT05578313	• Medical Cannabis	-	Recruiting
• Ulcerative Colitis				
• Pouchitis				
• Healthy Subject	NCT02439255	• Laboratory Biomarker Analysis	Not Applicable	Completed
• Tobacco Use Disorder		• Phytochemical		
		• Placebo		
		• Screening		
		• Questionnaire Administration		
Periodontal Diseases	NCT04705714	• Frankincense Extract	Phase 1	Completed
• Sleep Disorder	NCT05950932	• Melissa phytosome	Phase 4	Not yet recruiting
• Anxiety				
• Quality of Life				
Osteopenia	NCT03260803	• Oligopin	Phase 3	completed
Periodontal Diseases	NCT05138484	• Experimental group 10% mouthwash of *M. sylvestris* extract	Phase 3	Completed
Diverticulitis	NCT05596214	• Curcumin-Berberine (coptis)	Phase 2	Recruiting
• Immune System Tolerance	NCT05432362	• Aronia Juice	Not Applicable	Recruiting
• Depression				
• Obesity				
• Microbial Colonization				
• Diet, Healthy				
Gestational Diabetes Mellitus in Pregnancy	NCT05694520	• Pistachios consumption of 1.5 oz thrice per week	Not Applicable	Recruiting
• Markers of Inflammation	NCT05774613	• Effect of a Hibiscus sabdariffa beverage	Not Applicable	Recruiting
• Hyperglycemia, Postprandial				
• Hyperinsulinism				
Aging Problems	NCT04848792	• Sulforaphane	Not Applicable	Recruiting
Lung Cancer	NCT03232138	• Sulforaphane	Phase 2	Completed
Healthy Diet	NCT04329962	• Blueberry Powder Food Product	Not Applicable	Active, Not Recruiting
Nutrition Aspect of Cancer				
Cognitive Impairment				
Old Age; Debility	NCT06352099	• Dietary supplement with micronized diosmin, hesperidin and herbal extracts	Not Applicable	Not yet recruiting
Postoperative Atrial Fibrillation (POAF)	NCT05991700	• Freeze-Dried California Table Grape	Phase 1	Not yet recruiting
			Phase 2	
Myocardial Infarction	NCT03620266	• Bilberry	Not Applicable	Recruiting
		• Bioprocessed oat bran		
		• Combination bilberry/oats		
Insulin Resistance	NCT05717881	• Propolis	Not Applicable	Completed
Menopause	NCT03370991	Blueberry Powder	Not Applicable	Completed
Elevated Blood Pressure				
Hypertension				
Endothelial Dysfunction				
Metabolic Disease	NCT03685916	• Control	Not Applicable	Completed
		• Cumin Rice		
		• Cumin Drink		
		• Cornsilk Rice (Low dose)		
		• Cornsilk Rice (High dose)		
		• Cornsilk Drink (Low dose)		
		• Cornsilk Drink (High dose)		
		• Tamarind Rice		
		• Tamarind Drink		
Xerostomia	NCT06217614	Manuka honey-green tea	Not Applicable	Completed
Diabetes Mellitus				
Hypertension				
Post COVID-19 Condition				
Insulin Resistance	NCT04810572	Composition with Silymarin	Not Applicable	Completed
Inflammatory Bowel Diseases				
Overweight and Obesity		Low-mineral composition without Silymarin		
Healthy				

## 4 Overcoming Risks Related to the Direct Use of Plant-Based Crude Extracts in Medicine

Plant-based crude extracts, when used directly as drugs in medicine, pose several potential risks that require cautious consideration. One major concern is the variability of the chemical composition within plant extracts, which can differ based on factors such as geographical location, climate, and soil conditions.^49^ Plants contain a multitude of chemical compounds, and their concentrations vary, making it challenging to standardize dosages and to ensure consistent therapeutic effects. This variability can lead to unpredictable outcomes and fluctuating treatment efficacy. Another significant concern is the potential presence of toxic or harmful compounds in plant extracts. Although many plants have traditional medicinal uses, not all of their components are safe or therapeutically beneficial. Some constituents may cause adverse effects such as toxicity or allergic reactions. Without thorough purification and identification processes, there is an increased risk of including unwanted substances in medicinal preparations. Moreover, the absence of quality control and standardized production processes can raise contamination concerns. Plants can absorb heavy metals, pesticides, or other environmental contaminants that, if not properly monitored and addressed, may be present in crude extracts, posing serious health risks to patients. Additionally, the lack of regulatory oversight and standardized manufacturing practices for plant-based medicines can lead to inconsistent product quality, resulting in variable therapeutic effects and potential harm to patients. The direct use of a plant-based crude extract as a drug in medicine carries certain risks. It is important to conduct clinical trials to determine the safety, efficacy, and stability of these extracts before they can be used as crude drugs.^50^ Toxicity studies are crucial for determining the potential adverse effects of extracts. For example, research on the *Cistus ladaniferus L*. extract demonstrated that high doses of this extract caused mortality and toxicity symptoms in mice. In contrast, lower doses showed no adverse effects.^51^ The crude extract contains a mixture of compounds, including both active and inactive compounds, and the amount of bioactive compounds in the extracts is fairly low. Most attention has been given to curcumin, a turmeric derivative, as a key agent against a range of medical conditions, including microbial infection, angiogenesis, cancer, amyloidosis, etc., but due to its poor bioavailability, this compound has not been tested in clinical trials.^52^ In another study, oral administration of 1 g/kg of curcumin resulted in approximately 75% of the substance being eliminated through feces, with minimal detection in urine. However, when curcumin is administered intravenously or added to the perfusate of an isolated liver, it is actively transported into bile through concentration gradients of several hundred times.^53^ A significant portion of the administered drug, curcumin, was metabolized. Various studies have been conducted on enhancing the bioavailability of curcumin.

## 5 Bioactive Compounds and Mechanism in the Human Body

Bioactive compounds are vital for plants’ defense against pathogens, pests, and environmental challenges. Additionally, they enhance the plant’s color, taste, and scent, which makes them important for both culinary and medicinal applications. Among the most recognized bioactive compounds in plants are polyphenols, a group that includes flavonoids, phenolic acids, and tannins.^54^ Owing to their antioxidant effects, these compounds are associated with several health benefits, including a reduced risk of chronic conditions such as cancer, cardiovascular diseases, and neurodegenerative disorders.^55^ Alkaloids, which contain nitrogen and often have pharmacological effects in humans, are another important class of bioactive chemicals derived from plants. Known for their stimulating, addictive, and analgesic properties, notable alkaloids include caffeine, nicotine, and morphine. Another diverse class of bioactive substances found in plants is terpenoids, which support signaling, growth regulation, and defense. Some terpenoids, like essential oils and carotenoids, are useful in the food and cosmetic industries because of their antibacterial and antioxidant characteristics.^56^ Plant-derived bioactive compounds exhibit various mechanisms in the human body, including antitumor, anti-inflammatory, antidiabetic, and neuroprotective activities.^57^ These compounds interact with specific targets in cells such as proteins, nucleic acids, and membranes, influencing biological processes. Norio Kaneda discovered erypoegin K, an isoflavone that strongly induces apoptosis in human leukemia HL-60 cells. Additionally, Kaneda identified other compounds, including isoflavones, dimeric acridone alkaloids, carbazole alkaloids, and coumarin and quinoline derivatives, which possess apoptosis-inducing and anti-inflammatory properties.^58^ In vivo studies on prophylactic treatment using the beta-caryophyllene extract from black pepper demonstrated improved cognitive function. This effect was achieved by modulating the up-regulation of iNOS, bax, caspases, p-JNK, and p-38-MAPK induced by scopolamine. Additionally, treatment increased the functionality of bcl-2 and Trk B in the context of scopolamine-induced upregulation.^59^ Shoaib et al. published a detailed review on the bioactivity of plant-derived bioactive compounds and their effects on neurodegenerative disorders and mentioned various plants, such as Tilianin and Rosiridin from *Centella asiatica*, which have antioxidant, anti-inflammatory, antiaggregation, anticholinersterase and antiapoptotic properties.^60^ In wound healing, phytoconstituents scavenge free radicals, fight infections, and accelerate the healing process by promoting skin regeneration.^61^ Cannabinoids exhibit antiangiogenic properties by impeding the activation of the vascular endothelial growth factor (VEGF) pathway. This action inhibits angiogenesis and downregulates VEGF receptors 1 and 2. Cannabinoids also modulate various markers, including CB1, CB2, PSA, VEGF, IL-6, and IL-8, in human prostate cancer cell lines. Berberine acts as an antioxidant in melanoma cells by enhancing the activities of catalase and glutathione peroxidase enzymes. It mitigates oxidative damage by suppressing reactive oxygen species through the inhibition of the mTOR, PI3K, and AKT pathways, potentially reducing cancer risk.^62^ Triterpenoids, a type of terpenoid, modulate reactive oxygen species (ROS) levels and impact cell survival through various cell death modalities, influencing complex cell signaling pathways.^63^ They have also shown promise in cancer treatment due to their unique mechanisms and low side effects, with some progressing through clinical trials as anticancer agents.^64^ Curcumin modulates chemokines and receptors in the body. Curcumin exhibits anti-inflammatory, immunoregulatory, and antioxidative properties.^65^ Ying Liu et al. suggested that curcumin can act as an epigenetic regulator and exert a certain protective effect against manganese-induced damage to dopaminergic neurons.^66^ Curcumin is known to increase the expression of HO-1 mRNA and protein, potentially through its antioxidant effects. This action could activate cellular defense mechanisms against oxidative stress and lipid peroxidation products. In hepatocellular cancer, curcumin works through autophagy and apoptosis. Its capacity to trigger programmed cell death and regulate cellular self-digestion processes suggests a diverse strategy for preventing cancer progression. Furthermore, curcumin targets the a-class GST isozyme rGST8–8, which is involved in antioxidant defense. Curcumin enhances cellular detoxification processes and protects against lipid peroxidation products by modulating the expression of this enzyme.^67^ Bioactive compounds exert their effects through various mechanisms, including the modulation of gene expression, regulation of signaling pathways, and influence on cellular processes, thereby making them valuable for promoting health and addressing various diseases in the human body.

## 6 Techniques for the Extraction and Isolation of Bioactive Compounds

**Figure 1:**
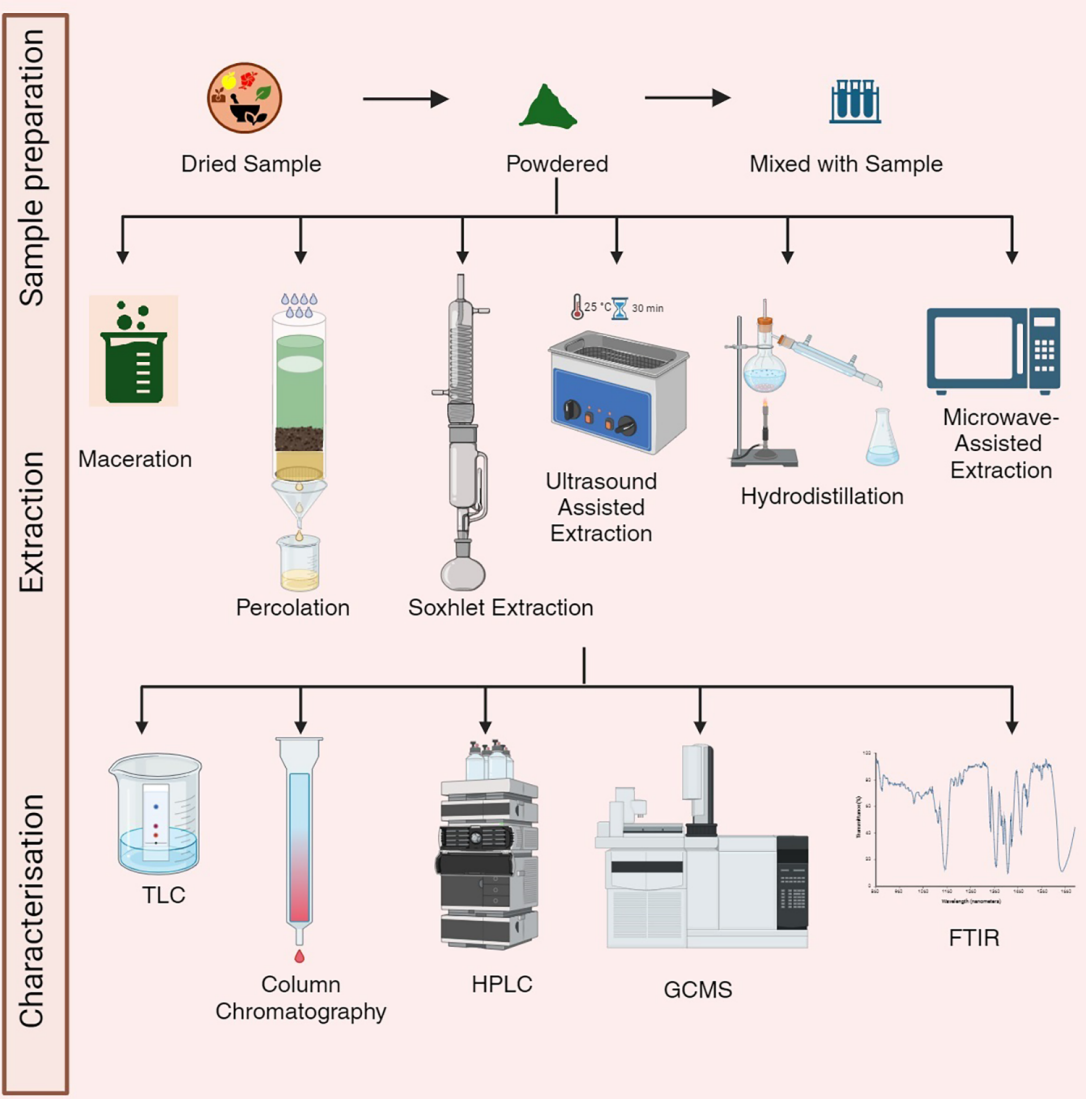
Flowchart showing the extraction, isolation, and characterization of natural products (generated using Biorender.com). Abbreviations: FTIR: Fourier transform infrared; GCMS: gas chromatography–mass spectrometry; HPLC: high-performance liquid chromatography; TLC: thin layer chromatography.

Historically, the extraction of bioactive compounds has been done using traditional methods that rely on simpler equipment and techniques compared to modern methods. Despite being less efficient and precise, these older techniques laid the foundation for today’s advanced extraction technologies. Usman et al. provides a comprehensive analysis of various traditional methods, including solid–liquid extraction (SLE), liquid–liquid extraction (LLE), and solid-phase microextraction (SPME). These older, conventional techniques come with several drawbacks, such as nutrient degradation, lengthy extraction durations, low efficiency, and high energy consumption. In contrast, modern and eco-friendly techniques like membrane ultrafiltration, surfactant-mediated extraction, supercritical fluid extraction (SFE), instant controlled pressure drop extraction, ultrasound-assisted extraction (UAE), microwave-assisted extraction (MAE) pressurized liquid extraction (PLE), and high-pressure technologies are now utilized.^68^ Maceration,^69^ percolation, distillation, Soxhlet,^70^ decoction,^71^ cold pressing,^72^ infusion, digestion extractions^73^ are the common conventional methods that has been used extensively in the past. As there are several extraction methods, there is no one single method that is regarded as standard for extracting compounds. There is no one technique that is thought to be the norm for extracting substances, because there are numerous extraction techniques. Before selecting a method, it is important to consider the drug’s type, the solvent, the product’s concentration, and its stability. Plant samples are full of intricate phytochemicals, making them challenging to separate; therefore, it is necessary to increase the polarity of the mobile phases to obtain better resolution for highly valuable separations. Thin-layer chromatography (TLC) has been used over time as a complementary technique, followed by column chromatography to identify the fractions of compounds produced by column chromatography. Thin layer and silica gel column chromatography (TLC) are methods that have been used for bioactive molecule separation and are accompanied by some analytical tools. The development of isolation techniques to efficiently obtain NPs in the pure form of complex crude extracts has become important. One approach involves the use of reversed-phase gradients from high-performance liquid chromatography (HPLC) to medium-performance liquid chromatography (MPLC) to separate pure compounds in milligram quantities^74^ (Figure 1). This strategy involves the prediction of retention behavior and resolution at the analytical scale to achieve consistent results at both the analytical and preparative levels. Another method is the application of hyphenated techniques such as LC/UV, LC/MS, and LC/NMR for screening chemicals and identifying the structures of compounds in raw plant extracts.^75,76^ These techniques provide rapid and online structural information, allowing for targeted isolation of compounds with novel or unusual features. Additionally, innovative extraction methods such as high hydrostatic pressure, ultrasound, supercritical fluid extraction, high hydrostatic assisted extraction, and different techniques have been investigated for the extraction of bioactive compounds from plant materials, offering greener and more efficient alternatives to conventional methods.^77,78^ Jha and Sit^78^ described the bioactive compounds extracted by different organic solvents. Although these are innovative methods for extracting and isolating bioactive compounds, more studies must be performed to improve the procedure and outcome.

## 7 Advances in the Study of Potential Therapeutics Using Nanotechnology

Nanomedicine is used in medical applications. Unlike traditional medicine, in which controlled release systems are used, nanotechnology can create novel drug delivery pathways, enhance drug absorption and usage, and improve drug targeting rates. Nanotechnology has shown great promise in the field of medicine, particularly in nanomedicine. This approach involves the use of nanocarriers to deliver drugs directly to target cells, increasing drug concentrations and minimizing side effects.^79^ Nanotechnology has also been applied in diagnostic instruments, targeted medicinal products, biomedical implants, and tissue engineering, improving the safety and efficacy of treatments.^80^ By enhancing compound solubility, influencing biodistribution, and managing drug release, nanomedicine has transformed the treatment of serious illnesses such as cancer, infections, and cardiovascular disorders.^81^ In the field of thrombosis therapy, this technique has been developed to accurately treat thrombi and improve antithrombotic therapy safety.^82^ Nanomaterial-based nanomedicines offer promise in antiviral therapy, presenting advantages over conventional treatment methods.^83^ Nanotechnology has notably influenced medicine by enhancing drug delivery, enhancing treatment effectiveness, and diminishing toxicity, thus setting the stage for future advances in nanomedicine.

### 7.1 Nanoformulation of Herbal Crude Extracts and Its Benefits

In recent years, there has been a growing emphasis on enhancing nanoformulations to improve the therapeutic effectiveness of medicinal drugs. The evolution of nanoformulations can be traced back to the early 2000s, when researchers began exploring nanocarriers to enhance the bioavailability, stability, and targeted delivery of phytochemicals.^84^ Nanocarriers such as liposomes, polymeric nanoparticles (PNPs), and solid lipid nanoparticles have been studied for their ability to encapsulate plant extracts and shield them from degradation, thereby enhancing their therapeutic potential.^85^ One of the main motivations behind the creation of plant extract nanoformulations is to overcome the shortcomings of conventional herbal remedies, such as their uneven bioavailability and inability to provide targeted administration.^86^ Using crude plant extracts to formulate nanostructures offers several advantages. Plants are used to synthesize nanoformulations by combining the corresponding salts with plant extracts. This reaction mixture undergoes a redox reaction, and the formation of nanoparticles is indicated by the change in color. Typically, the process of creating nanoparticles using plant extracts entails giving electrons to the metal ions, which result in the production of the particles. When processed metal ions are transformed from mono- or divalent oxidation states into their elemental form (zerovalent) during the first activation phase of nanoformulation biosynthesis, the reduced metal atoms are nucleated. Heat plays a crucial role in the reaction, as smaller nanoparticles rapidly agglomerate to form larger, thermodynamically more stable nanoparticles. Moreover, the reduction of metal ions occurs, and additional growth processes produce nanoparticles with a variety of shapes and sizes, including spheres, cubes, hexagons, rods, and wires.^87^

Their ability to penetrate the skin more effectively and release active chemicals into the skin makes them a viable instrument for the creation of cosmeceuticals based on plant extracts. In addition, for the manufacturing of vaccine adjuvants such as *Quillaja* saponins, nanoformulations provide an economical and sustainable substitute for conventional purifying techniques.^88^ Furthermore, nanotechnology-based drug delivery strategies have been employed to overcome the limitations of native phytocannabinoids in medical cannabis applications.^89^ Nanoformulation systems, including lipid-based nanoparticles and cyclodextrins, have shown promise for improving the pharmacokinetic profile and the targeted delivery of phytocannabinoids. Moreover, nanoformulations can reduce toxicity to healthy cells by encapsulating bioactive compounds within nanoparticles while maintaining their efficacy against target cells or pathogens. This targeted delivery reduces the risk of adverse side effects and offers a controlled release. Overall, the development of targeted delivery systems using nanoformulations from crude plant extracts holds great potential for enhancing the therapeutic applications of plant-based medicines and bioactive compounds. The different methods of synthesis of nanoformulations are presented in Table 2. These nanoformulations have the potential to offer safer, more effective, and more targeted treatments for a wide range of diseases while also preserving the traditional knowledge of herbal medicine.

**Table 2:** Different Methods for the Synthesis of Nanoformulations

**Types of nanoformulations**	**Method of synthesis**	**Benefit**
Dendrimers	Divergent growth method	- Allows the incorporation of various functional groups at different positions within the dendrimer structure, ensuring a well-defined and uniform product.^90^
		- It often involves less expensive reagents, making the process cost-effective.
	Convergent method	- This method allows for the construction of structurally well-defined macromolecules with specific properties and allows easy purification of the product.^91^
Polymeric nanoparticles	Dispersion of preformed polymers	- Requires low energy.
		- Can generate nanoparticles with higher concentration compared to other techniques.
		- Single step method.^92^
	Supercritical fluid technology	- Suitable for industrial-scale production.
		- Uses solvent that is environmentally friendly and nontoxic/carcinogenic.^93^
	Polymerization of monomers	- Enhances target specificity and safety of nanoparticles.
		- Enhances stability of core-polymerized shell nanoparticles.
		- Allows modification of properties and structural changes for nanoparticles.^94^
Liposomes	Reversed-phase evaporation	- Simple to perform
		- Encapsulates water-soluble material.^95^
	Detergent removal	- It allows for the preparation of proteoliposomes for studying membrane proteins.
		- Compatible with sensitive payloads like proteins, peptides, and nucleic acid^96,97^
	High-pressure extrusion	- High Biocompatibilty
		- Low toxicity
		- Controlled disturibution and ability to perform targeted, extended and sustained release.^98,99^
Nanoemulsions	Dispersion	- Allows the uniform particle size distribution.
		- Have the ability to penetrate the skin.^100^
	Ultra Sonication	- Breaks down the particle size to the nanometric range.
		- It can give a surfactant-free nanoemulsion.^101^
	Phase inversion temperature	- Requires low energy
		- Widely used in industries
		- Forms small droplet sizes
		- High stability^102^
	Spontaneous emulsification	- Energetic yield optimized by chemical instability
		- Potential for industrial scale-up.
		- Preserves fragile compounds.^103^
Micelles	Dialysis	- Allows the separation of organic compounds used in their formation.
		- Exhibits excellent biocompatibility and stability.^104^
	Oil in water	- Biocompatible water-in-oil microemulsions solubilize hydrophobic compounds efficiently.
		- Engineered for controlled release of encapsulated compounds, demonstrating biocompatibility.^105^

The use of NP-driven nanoformulations to treat burns, infections, diabetes, cancer, and other human disorders is an emerging field. The vast majority of current plant-based medicine research has concentrated on therapeutically potent phytoconstituents rather than new formulations. However, in the last few decades, scientists have made great strides toward creating “novel drug delivery systems” (NDDSs) that improve the effectiveness of therapy and minimize the undesirable effects of bioactive molecules. Bioactive compounds and plant extracts have been used to create a variety of novel therapeutic formulations, including nanocapsules, polymer micelles, liposomes, nanogels, phytosomes, nanoemulsions, transferosomes, microspheres, ethosomes, injectable hydrogels, PNPs, dendrimers, and others.^106^ Recent investigations over the past few years are shown in Table 3.

**Table 3:** Different Types of Nanoformulations with Plant Extracts Have Been Used in Different Studies in Recent Years^a^

Plant	Part of plant	Type of Nanoformulation	Study Model	Reference
*Lasiurus scindicus* and *Panicum turgidum*	Seed	AgNPs	Metastatic breast cancer monolayer cells	(107)
*Perilla frutescens*	Leaves	AgNPs	*Escherichia coli* and *Staphylococcus aureus; Candida albicans*; CF-7 cancer monolayer cells	(108)
*Evodia rutaecarpa*	Fruit	EVO encapsulated BSA nanoparticles	Breast carcinoma cell monolayer lines	(109)
*Nyctanthes arbortristis*	Flower	ZnO-NPs	Lung and Cervical Cancer cells	(110)
Shilajit	Himalayan Rock	ZnO-NPs	HeLa cell line and Cervical Cancer cells	(111)
*Justicia adhatoda*	Leaves	AgNPs	A549 Cells	(112)
Pea protein and Curcumin	Rhizome	(PPI-Cur) nanoparticle	HepG2 cells	(113)
*Psidium guajava*	Leaves	(DA/CMC/TiO_2_) NP with leaf extract	MG-63 cells	(114)
*Cassia fistula*	Leaves	Zinc oxide-copper oxide nanoparticles	Panc-1 and OVCAR-3 cancer cells	(115)
*Pedalium murex* L	Fruit	CuNPs	A594 Cells	(116)
*Centella asiatica*	Leaves	Nanoparticle	For antimicrobial and antioxidant property	(117)
*Jatropha dioica*	Root	PNP	Vero cells and HSV-1 strain and HSV-2 strain	(118)
*Cassia fistula*	Flower	AgNP	Antimicrobial and anticancer	(119)
Plantago major	Leaves	Nanofiber	Antimicrobial study of wound	(120)
*Glycine max* L	Seed	Gold nanoparticles	Osteoporosis in male rats	(121)
*Eucalyptus tereticornis*	Leaves	Polymeric nanoparticle Nanoemulsion	Type 2 diabetes mellitus in mice model	(122)
*Coriandrum sativum L*	Fruit	Lipid nanostructure carriers	Antiaging activity of Swiss Albino mice	(123)

a Abbreviations: AgNPs: Silver nanoparticles; A549: Lung adenocarcinoma cells; B16F10: Malignant murine melanoma cell line; CuNPs: Copper nanoparticle; CMC: Carboxymethyl cellulose; DA: Dopamine; EVO: Evodiamine; HSV-1, HSV-2: Herpes simplex virus type 1 and type 2; MG-63: Human osteoblastic line; PPI-Cur: Pea protein isolate-curcumin; PNP: Polymeric nanoparticle; PANC-1: Human pancreatic cell line; OVCAR-3: Cisplatin refractory cell line; TiO_2_: Titanium oxide; ZnO NP: Zinc nanoparticle

Concerning the choice of nanoformulation, multiple factors are kept in mind depending on the specific application, targeted delivery, bioavailability, solubility, and stability of the active ingredient, and there is no one-size-fits-all answer to which type of nanoformulation is better. Each has its advantages and disadvantages. In the development of nanodrugs, it is imperative that the formulation facilitates the transportation of the drug from the site of administration to the site of action while also providing protection against environmental factors such as pH changes, enzymatic degradation, and biochemical breakdown.^124^ One of the emerging techniques is encapsulation, which can help maintain the biological activities and bioavailability of bioactive compounds.^125^ These formulations enable the incorporation of active ingredients from medicinal plants into carrier systems that traditional formulations cannot accommodate, thus improving the delivery of therapeutic compounds at appropriate doses and rates.^126^ Additionally, organometallic complexes containing NPs have shown enhanced cytotoxicity toward cancer cells when encapsulated in nanoformulations, leading to improved anticancer potency and reduced toxicity.^127^ Overall, nanoformulations offer a promising approach to optimizing the therapeutic potential of NPs in medicine.

### 7.2 Translation Approach of Nanoformulation

Translational research means that knowledge from basic sciences is translated for further processes of drug development, which, in turn, can create a new drug or a device that is beneficial for clinical purposes or even commercialization. The translation of nanoformulations necessitates the scale-up of the synthesis and processing to achieve precise control over nanoscale properties. This includes ensuring reproducibility in terms of size, polydispersity, safety, quality, and drug efficacy.^128^ The clinical translation of nanomedicines faces several challenges, including practical and clinical feasibility, preclinical and clinical aspects, and pharmaceutical considerations.^129^ Despite the progress in nanomedicine research, only a small percentage of basic science research has successfully translated into clinical applications.^130^ Nanotechnology-based formulations have gained importance in cancer prevention and treatment, with various types of nanoformulations being investigated for different cancer types.^131^ Liposomes, polymeric micelles, and nanoparticles are the main nanocarrier platforms used in cancer nanomedicines, with several formulations already on the market and in clinical development.^132^ Passive targeting through enhanced permeability and retention is the most common approach, but active targeting strategies are still under development. Nanotheranostics, which combine diagnostic imaging agents and pharmacological moieties in one carrier platform, offers a promising approach for improving drug delivery to the brain.^128^ Nanomedicine aims to shift the balance from possibly harmful to beneficial treatment. To successfully address issues with solubility, stability, biodegradation, and other issues during in vivo application, traditional nanomedicines that have been approved for use in disease management are essential for delivery system. These issues are largely resolved by optimizing the pharmacokinetics and biodistribution of loaded compounds through regulation of their physical and chemical properties. Nevertheless, these strategies fall short of fully utilizing the medicinal potential of drugs. Therefore, to boost their therapeutic efficacy, next-generation nanomedicines with complex activities that go beyond delivery modalities must be developed.^133^ Many studies have been performed on the application of NPs, and nanomedicine to enhance the therapeutic index and safety of nanoparticles (NPs) in treating inflammatory conditions, including those affecting the nervous system, intestines, bones, and eyes.^134^ For instance, the successful encapsulation of curcumin and its structural analogues (diferuloylmethane and dibenzoylmethane) has been achieved using nanoemulsions.^135^ In phase I human clinical studies, these medicines exhibited significant therapeutic potential with reduced toxicity. Site-specific delivery and enhanced efficacy of bioactive compounds are achieved through the loading of phytochemicals with bioactive properties into nanoparticles. Another benefit is that some nanoparticles gradually deliver bioactive phytochemicals into cells, which helps to maintain therapeutic benefits. Among all the bioactive compounds, curcumin is the most extensively studied bioactive compound nanoparticle and has gone through a thorough phase trial. Other natural compound-based nanoparticles have also undergone trials (Table 4). Although studies are being conducted, more research has to be done to determine the efficacy and mechanism of nanoparticles as the amount of bioactive compound used for animal testing is not enough for the human body.

**Table 4:** Clinical Trials on Nanoformulations Based on NPs for the Treatment of Different Diseases^a^

Condition Targeted	Drug derived from	NCT Number	Intervention	Study Model	Phase
Otomycosis	*Moringa oleifera*	NCT04768829	*Moringa oleifera* leaf 10 mg/100 mL	Human	Early Phase 1
Dental Caries	Clove	NCT04390256	Clove water extract	Human	Not Applicable
Dental Carries	*Pelargonium graveolens*	NCT05816512	• Gold nanoparticle from *Pelargonium graveolens*	Human	Not applicable
Gingivitis			• Chlorhexidine gluconate mouthwash		
Periodontitis					
Carcinoma, Non-Small-Cell Lung	Paclitaxel	NCT02716038	• MPDL3280A	Human	Phase 2
			• Carboplatin		
			• Nab-paclitaxel		
Recurrent Aphthous Ulcer	Curcumin	NCT04385979	Gel	Human	Completed
Recurrent Aphthous Stomatitis					
Oral Cancer	Quercetin	NCT05456022	• Quercetin 3,3′,4′,5,6-Pentahydroxyflavone, 2-(3,4-Dihydroxyphenyl)-3,5,7-trihydroxy-4H-1-benzopyran-4-one	Cell line	Phase 2
			• Quercetin-encapsulated PLGA–PEG nanoparticles (Nano-QUT)		
			• Doxorubicin chemotherapeutic drug as a positive control		
Schizophrenia	Curcumin	NCT02104752	Curcumin	Adult Human	Phase 1
Cognition					Phase 2
Psychosis					
Traumatic Pulp Exposure in Children	Curcumin	NCT06029023	MTA Cement, propolis nano particle, curcumin nanoparticles	Children	Phase 1
Breast cancer	Doxorubicin	NCT03749850	• LTLD Procedure: MR-HIFU induced hyperthermia	Human	Phase 1
			• Cyclophosphamide		

a Abbreviations: LTLD: Lyso-thermosensitive liposomal doxorubicin; MDPL3280A: Anti-PD-L1 monoclonal immunoglobulin-G1 antibody; MTA: Mineral trioxide aggregate; MR-HIFU: Magnetic resonance imaging-guided high-intensity focused ultrasound; Nano-QUT: Nanoparticles loaded with quercetin; PEG: Polyethylene glycol; PLGA: Poly lactic-*co*-glycolic acid.

### 7.3 Natural Product-Based Nanoformulations for the Treatment of Various Diseases

NP-based nanoformulations have emerged as promising agents for treating diseases such as atopic dermatitis (AD), microbial infections, diabetes mellitus, cancer, and neurodegenerative disorders. For diabetes management, several well-known herbs have demonstrated the potential to lower blood glucose levels, offering the possibility of improved glycemic control or reduced insulin injections, which is a desirable outcome. However, the selection of plants for treatment can be influenced by factors such as the stage of diabetes progression, coexisting medical conditions, availability, cost, and safety considerations. Recently, a study investigated the potential effects of topical application of a rutin nanoformulation on wound healing in streptozotocin (STZ)-induced hyperglycemic rats treated with metformin. This study focused on the nanoformulation’s anti-inflammatory and antioxidant properties.^136^ Another recent study showed that a carvacrol-nanostructured lipid carrier showed better antioxidant, anti-inflammatory, and antibacterial activity and helped improve wound healing in diabetic mice.^137^ NP nanoformulations have shown promise in diabetes management. Studies have explored nanoformulations of natural compounds such as myricetin encapsulated in chitosan nanoparticles,^138^ as well as nanocurcumin compounds with enhanced bioavailability for antidiabetic effects and for managing diabetic complications.^139^ Additionally, photosynthesized nanoparticles produced from plant extracts have been studied for their potential for use in diabetes therapy, as they are more effective and safer than conventional nanoparticles.^140^ Herbal nanoformulations have been developed to address the limitations of poor stability and poor absorption of herbal remedies in managing type 2 diabetes mellitus, resulting in improved biological properties in both in vitro and in vivo models.^141^ These NP nanoformulations promise to provide alternative and potentially more effective treatments for diabetes. In addition to treating diabetes, the nanoformulation of NPs has shown therapeutic potential in atopic dermatitis.^142^ Tacrolimus-loaded polycaprolactone nanocapsules have shown success in controlling AD, offering improved drug release and anti-inflammatory activity.^143^ Recently, another study involving the topical use of clove-oil-based nanomicelles for treating atopic dermatitis caused by bacterial infection was conducted. In vitro studies have shown that these drugs are more effective than conventional drugs for treating such disorders.^144^ PNPs made from natural polymers have also been explored for topical drug delivery in various skin diseases, including AD, showing improved drug stability, controlled release kinetics, and enhanced therapeutic efficacy.^145^ There are few reviews or studies on atopic dermatitis caused by nanoformulations of different plant extracts, which shows the potential of this medicine for treating skin disease.^146−148^ NPs are emerging as promising antimicrobial agents, offering an alternative to traditional antibiotics. Pathogenic microorganisms have been effectively targeted using medicinal herbs and nanosilver. Herbal medicines are favored in healthcare because of their cost-effectiveness and abundance of antibacterial compounds.^149^ Nanoformulations based on plant extracts have shown promising antibacterial properties. Different studies have focused on utilizing plant extracts such as *Scinus areira* essential oil,^150^*Rhazya stricta root extract*,^151^*dandelion*,^152^*curcumin*,^153^*Onopordum acanthium* extract,^154^*Phyllanthus emblica* plant extract,^155^*Prunus spinosa* berries,^146^ and *Cassia fistula*.^119^ Chang et al. provided a detailed review of the mechanism by which polyphenols act as antibacterial agents and investigated the use of nanoformulations for the delivery of polyphenols to different nanoparticles, such as polymer-based NPs, metal-based NPs, lipid nanoparticles, and various types of nanoscaffolds.^153^ A review by Aida et al.^156^ noted that nanoformulations containing carvacrol and thymol exhibited antimicrobial properties and demonstrated immunostimulant effects. These formulations were also found to promote the growth of bifidobacterial gut species known for enhancing immune system function.^157,158^ Recently, an in vitro study was conducted on mouthwash containing zinc oxide nanoparticles, a chamomile and green tea formulation tested for its antibacterial effects.^159^ Nanoformulations of these NPs enhance their efficacy and delivery in antifungal studies. For instance, nanoemulgels containing timur oil and rosemary oil demonstrated significant antifungal effects against *Candida albicans*.^160^ Additionally, nanoliposomes loaded with clove essential oil and tea tree oil exhibited high entrapment efficiency and antifungal activity against *Trichophyton rubrum* fungi.^161^ Essential oils and nanocarrier formulations show promise for antifungal studies in vaginal candidiasis treatment, enhancing efficacy through phytoconstituents and altered characteristics.^162^ Using flavonoids, terpenes, and quinones, NP-based nanomedicine enhances the antifungal treatment efficacy. Nanotechnology has improved drug delivery and selectivity, with promising future advancements in antifungal therapy.^163^ These NP-based nanoformulations offer a safe and effective approach to combating fungal infections, especially in cases of resistance to conventional antifungal therapies. Additional research and clinical trials are crucial to validate the therapeutic potential and safety of these nanoformulations for antifungal treatment. Despite the broad spectrum of anticancer properties exhibited by natural compounds in numerous in vitro preclinical settings, there have been challenges in translating these promising results to in vivo systems, often resulting in unmet expectations. There are numerous clinical trials involving curcumin,^164−166^ but its use as the sole drug for cancer treatment is lacking. By utilizing nanotechnology, NPs such as curcumin, epigallocatechin gallate (EGCG), resveratrol, and genistein can be encapsulated in nanoparticles, liposomes, or micelles to enhance their delivery to target cancer sites.^167^ Phytochemical-based nanoformulations have improved cytotoxicity in various cancer cell lines, including breast, glioma, cervical, and colorectal cancer.^168,169^ These nanoformulations exhibit dual functionality, demonstrating both the inhibition of cancer cell growth and the potential to enhance the efficacy of traditional chemotherapeutic agents synergistically. This dual action offers a promising strategy for overcoming drug resistance and improving outcomes in cancer treatment. In the context of neurodegenerative disorders, a primary challenge for most medicinal substances is their inability to traverse the blood-brain barrier (BBB). This barrier presents a substantial impediment to the development of treatments for neurodegenerative disorders.^170^ Yan Dou et al. used quercetin, a photooxidant albumin nanoagent, and tested its ability to treat advanced Alzheimer’s disease, as it has neuroprotective effects and potential multitargeting mechanisms in nanomedicine.^171^ Another study was performed with curcumin-loaded selenium PLGA nanoparticles targeting amyloid plaques in vivo to observe the effects of decreasing Aβ plaque aggregation and decreasing inflammation on AD pathology.^172^ Essential oils are also being employed for nanoparticle creation, and their incorporation into neural systems has shown enhanced efficacy in combating various diseases.^173^ Studies have investigated the synthesis of gold, silver, and zinc oxide nanoparticles using extracts or organisms from *Terminalia arjuna, Gloriosa superba, Aquilegia pubiflora*, and *Aspergillus austroafricanus* for potential therapeutic applications in neurological disorders.^174^ Likewise, many studies on different diseases and disorders are being performed. It is important to highlight that these nanoformulations require further intensive research, and clinical trials are essential to fully validate their therapeutic potential and safety for clinical applications.

## 8 Conclusion and Perspective

Natural products (NPs) hold significant pharmacological importance globally and are relatively accessible. However, due to their complex structures, synthesizing them poses a considerable challenge, and extraction processes are often not straightforward, cost-effective, or convenient. Despite being so popular among locals, the limitations still remain over the synthetically synthesized drug. The translational research of NPs against various diseases shows their potential as a great candidate for drug development. However, to make this process much more successful, combining it with nanotechnology would bring the best out of NPs through the enhancement of targeted therapy, low side effects, and good bioavailability, which are the major drawbacks of simply using the crude extract. NP-based nanoformulations are a new field of research that requires a thorough understanding of the synthesis and the mechanism underlying this approach. This approach can greatly increase the extent to which the size of the nanoparticle can be altered for diagnosis, imaging, and controlled treatment. Combining NPs with different nanocarriers can solve most of the challenges that they face. This review outlines the history of natural products (NP), emphasizes the need for clinical trials to evaluate the safety, effectiveness, and stability of plant-based crude extracts before they can be utilized as medications, discusses the difficulties in isolating complex phytochemicals from plant materials, and highlights major advancements in creating new drug delivery systems.

This review is based on recent research on NPs and nanoformulations, which means that intensive studies must be performed on this topic, as it is still under investigation, and the focus must be on making clinically approved nanoformulations. Different plants have different bioactive compounds, and they act differently against diseases. If more attention is given, the techniques may improve, and more problems related to the unavailability of the compound can be solved. Most of the nanoparticles formed are in the form of metal nanoparticles, which are known to have few drawbacks. Their cytotoxic nature limits their use in biomedical applications, where biocompatibility is crucial, whereas micelles and polymeric nanoparticles are quite compatible with medical applications. The use of polymers such as PLGA, that degrades into lactic acid and glycolic acid, which are both naturally occurring compounds, reduces the toxicity during degradation. Every step of the making process, especially degradation, needs a special focus so that researchers do not face these challenges during the trials.
